# Ovulation-inducing factor: a protein component of llama seminal plasma

**DOI:** 10.1186/1477-7827-8-44

**Published:** 2010-05-12

**Authors:** Marcelo H Ratto, Wilfredo Huanca, Gregg P Adams

**Affiliations:** 1 Faculty of Veterinary Sciences, Universidad Austral de Chile, Valdivia, Chile; 2 Laboratory of Animal Reproduction, Universidad Mayor Nacional de San Marcos, Lima, Peru; 3 Department of Veterinary Biomedical Sciences, University of Saskatchewan, Saskatoon, Canada

## Abstract

**Background:**

Previously, we documented the presence of ovulation-inducing factor (OIF) in the seminal plasma of llamas and alpacas. The purpose of the study was to define the biochemical characteristics of the molecule(s) in seminal plasma responsible for inducing ovulation.

**Methods:**

In Experiment 1, llama seminal plasma was centrifuged using filtration devices with nominal molecular mass cut-offs of 30, 10 and 5 kDa. Female llamas (n = 9 per group) were treated i.m. with whole seminal plasma (positive control), phosphate-buffered saline (negative control), or the fraction of seminal plasma equal or higher than 30 kDa, 10 to 30 kDa, 5 to 10 kDa, or < 5 kDa. In Experiment 2, female llamas (n = 7 per group) were given an i.m. dose of seminal plasma treated previously by: 1) enzymatic digestion with proteinase-K, 2) incubation with charcoal-dextran, 3) heating to 65°C, or 4) untreated (control). In Experiment 3, female llamas (n = 10 per group) were given an i.m. dose of pronase-treated or non-treated (control) seminal plasma. In all experiments, llamas were examined by transrectal ultrasonography to detect ovulation and CL formation. Ovulation rate was compared among groups by Fisher's exact test and follicle and CL diameters were compared among groups by analyses of variance or student's t-tests.

**Results:**

In Experiment 1, all llamas in the equal or higher than 30 kDa and positive control groups ovulated (9/9 in each), but none ovulated in the other groups (P < 0.001). In Experiment 2, ovulations were detected in all llamas in each treatment group; i.e., respective treatments of seminal plasma failed to inactivate the ovulation-inducing factor. In Experiment 3, ovulations were detected in 0/10 llamas given pronase-treated seminal plasma and in 9/10 controls (P < 0.01).

**Conclusions:**

We conclude that ovulation-inducing factor (OIF) in llama seminal plasma is a protein molecule that is resistant to heat and enzymatic digestion with proteinase K, and has a molecular mass of approximately equal or higher than 30 kDa.

## Background

In his monograph of *The Biochemistry of Semen *nearly 50 years ago, Thaddeus Mann summarized the natural properties of seminal plasma as a vehicle for sperm transport, a controller of sperm motility and capacitation, and as a stimulant of uterine contractility [1]. In light of recent studies, however, we now know that the role of seminal plasma also includes effects on ovarian function in the inseminated female. Intramuscular or intrauterine deposition of llama or alpaca seminal plasma induced ovulation in females of both species [2] - species classified as reflex or induced ovulators [3,4]. Results support the hypothesis that the ovulation-inducing factor (OIF) in seminal plasma effects ovulation via a systemic rather than a local route since 1) intramuscular administration of seminal plasma in female llamas and alpacas resulted in a surge in circulating concentrations of LH within 15 minutes of treatment [2], 2) disruption of the endometrial mucosa by curettage facilitated absorption of OIF and increased the ovulatory effect of seminal plasma given by intrauterine infusion, and 3) ovulation was not associated with physical stimulation of the genital tract [5].

Results of studies in South American camelids are consistent with early studies in Bactrian camels in which ovulation was induced by intravaginal semen deposition without any physical contact with the male [6]. Authors of studies on human seminal plasma reported the presence of molecules that immuno-reacted with GnRH antibodies [7,8], but it is unknown if these immuno-reactive molecules can induce ovulation in humans. Based on LH secretion from primary cultures of rat pituitary cells [9], the putative ovulation-inducing factor in alpaca seminal plasma had GnRH-like activity but was not GnRH since its biological activity was not suppressed when anti-GnRH antibodies were added to the culture medium.

The findings of studies done in camelids have implications that extend beyond camelid species. Ovulation-inducing factor has been detected in the seminal plasma of species that are not classified as induced ovulators; i.e., bull [10], stallion and boar [11]. The role of OIF in cattle has not been determined, but the preovulatory LH surge was advanced in cows when mating was done during the first 6-8 hours of behavioral estrus versus later [12]. As well, intrauterine deposition of boar seminal plasma accelerated ovulation in gilts [13]. Whether these factors are related to the ovulatory mechanism in humans or other species, and whether these factors are related to those of camelids, remains unknown.

To date, the biochemical composition of OIF, as a single or complex of elements, has not been identified. The purpose of this study was to provide preliminary biochemical characterization of the molecule(s) present in the seminal plasma of llamas responsible for inducing ovulation in this species. By attempting to inactivate or exclude the bioactive fraction of seminal plasma, the specific objectives were to 1) determine the approximate molecular mass cut-off fractions that are associated with a loss of bioactivity (Experiment 1), 2) determine the bioactivity of llama seminal plasma after enzymatic digestion with proteinase K, filtration with charcoal dextran, or heat treatment (Experiment 2), and 3) determine the effects of more aggressive enzymatic digestion with pronase E on the bioactivity of OIF (Experiment 3).

## Methods

### Experiment 1 - molecular mass cut-off

The experiment was conducted during February to March at the Quimsachata Research Station in the Department of Puno, Peru (15°S, 71°W, and 4,500 m above sea level). Semen was collected from 3 llamas using a phantom mount fitted with an artificial vagina [2]. Ejaculates were diluted 1:1 (v/v) with phosphate buffered saline (PBS, Gibco, Grand Island, N.Y., USA) and centrifuged for 30 minutes at 1500 × g. The supernatant was decanted from the spermatozoa and a drop was evaluated by microscopy to confirm the absence of cells. If spermatozoa were detected, the sample was centrifuged again in like manner. Sperm-free seminal plasma was stored at -70°C. At the time of use, the stored seminal plasma was thawed and ejaculates were pooled within and among males. A 15 ml volume of pooled seminal plasma was fractionated in a sequential manner using centrifugal filter devices with nominal molecular mass limits of 30, 10, and 5 kDa (Amicon Ultra-15, Millipore Corporation, Bedford, MA, USA). The 15 ml sample was initially loaded into a 20 ml filter device with a molecular mass cut off of 30 kDa and centrifuged at 2500 g for 45 minutes at 4°C. After centrifugation, the retained volume (20 ul) was re-suspended to the original volume of 15 ml with PBS supplemented with 50 μg/ml of kanamycin (Sigma Chemical Co., St Louis, MO, USA) and stored at -20°C as the ≥ 30 kDa fraction of seminal plasma. The portion of seminal plasma that passed through the 30 kDa filter was subsequently loaded into a filter device with a molecular mass cut off of 10 kDa and was centrifuged in like-manner. Again, the retained volume after centrifugation was re-suspended to the original volume of the sample (15 ml) and stored as the 10-30 kDa fraction of seminal plasma. The remaining filtrate was loaded into a filter device with a molecular mass cut off of 5 kDa, and centrifuged as described. Both the retained portion as well as the filtrate were re-suspended to the original sample volume (15 ml), as described, and stored as the 5-10 kDa and < 5 kDA fractions, respectively. Thus, the process yielded four fractions of seminal plasma based on approximate molecular mass (i.e., ≥ 30 kDa, 10-30 kDa, 5-10 kDa, and < 5 kDa).

Mature non-lactating female llamas (n = 60), ≥ 4 years of age and weighing an average of 120 kg, were examined daily by transrectal ultrasonography (Aloka SSD 500, Tokyo, Japan) using a 7.5 MHz linear-array transducer. Llamas were selected (n = 54) when a growing follicle of ≥ 8 mm in diameter was detected (i.e, capable of ovulating) [14], and then assigned randomly to 6 groups (n = 9 per group). In the respective groups, llamas were given an intramuscular dose of 1.5 ml of whole seminal plasma (positive control), or the seminal plasma fraction ≥ 30 kD, 10 to 30 kD, 5 to 10 kD, or ≤ 5 kD, or PBS (negative control). Llamas were examined daily by transrectal ultrasonography until Day 2 (Day 0 = treatment) to detect ovulation, and again on Day 8 to detect the presence of a corpus luteum (CL). Ovulation was defined as the sudden disappearance of a large follicle (≥ 8 mm) that was detected during the previous examination, and was confirmed by subsequent detection of a CL [14,15].

### Experiment 2 - treatment with proteinase K, charcoal, or heat

The experiment was conducted from October to November at the University of Saskatchewan, Canada (52° N, 106° W and 500 m above sea level). Semen was collected from 4 llamas by artificial vagina over a period of 2 months (22 ejaculates per animal), and was processed as described above. A volume of 20 ml of seminal plasma was prepared for each of 4 treatments: 1) enzymatic digestion with proteinase-K (Promega Biosciences Inc, San Luis Obispo, California, USA), 2) incubation with charcoal-dextran, 3) heating to 65°C, or 4) untreated (control). Enzymatic digestion involved incubation with 500 μg/ml proteinase-K in a shaker water bath at 38°C for 1 hour. The enzymatic reaction was terminated by adding phenylmethylsulfonyfluoride (PMSF, Sigma-Aldrich, St. Louis, Missouri, USA) to a final concentration of 5 mM. Samples were centrifuged at 1500 × g for 5 minutes and the supernatant was aspirated and stored at -70°C. Incubation with charcoal dextran (0.12 g/l: 12 g/l, Sigma-Aldrich, St Louis, Missouri, USA) was done at 4°C overnight, and charcoal was removed by centrifugation at 7500 × g for 15 minutes followed by filtration through a series of 0.45-μm filters and finally through a 0.22-μm filter (Millipore Corporation, Bedford, MA, USA), and stored at -70°C. Heat treatment involved incubation in shaker water bath at 65°C for 10 minutes or 1 hour. The sample was centrifuged at 1500 × g for 5 minutes, and the supernatant was aspirated and stored at -70°C. For the control group, seminal plasma was incubated in a shaker water bath at 38°C for 1 hour without the presence of proteinase-K, and PMSF was added to a final concentration of 5 mM. The sample was centrifuged at 1500 × g for 5 minutes and the supernatant was aspirated and stored at -70°C.

Mature non-lactating female llamas (n = 30), ≥ 4 years of age and weighing 100-150 kg, were given 5 mg Armour Standard LH (Lutropin-V, Bioniche Animal Health, Belleville, ON, Canada) to synchronize follicular wave emergence among animals [16]. Twelve days after LH administration, llamas with a follicle ≥ 8 mm in diameter were assigned randomly to 4 groups (n = 7 per group) and given an intramuscular dose of 2 ml of seminal plasma from the respective treatments. Llamas were examined daily by transrectal ultrasonography until Day 2 (Day 0 = treatment) to detect ovulation, and again on Day 8 to detect the presence of a CL.

To examine the effects of treatment on seminal plasma protein band profiles, samples from each treatment were reduced, denatured, and separated by electrophoresis on 12% polyacrylamide gel (SDS-PAGE) based on the protocol of Laemmli [17]. Gels were stained with Coomassie Blue R-250 (Sigma-Aldrich, St Louis, Missouri, USA).

### Experiment 3 - treatment with pronase E

The experiment was conducted from May to June at the University of Saskatchewan, Canada. Llama seminal plasma (10 ml), collected as described in Experiment 2, was incubated with pronase E (Sigma-Aldrich) at a final concentration of 500 μg/ml, in a shaker water bath at 38°C for 1, 3, 6, 9, or 12 hours (2 ml seminal plasma sample/time). A sample from each incubation time was subjected to protein separation by SDS-PAGE, as described in Experiment 2. Based on the degree of enzymatic digestion indicated by protein band patterns in the electrophoresis gel, the 12-hour incubation period was selected for the purposes of the following llama bioassay. A larger volume of llama seminal plasma (20 ml) was incubated with pronase E for 12 hours, as described, and the reaction was terminated by adding PMSF to a final concentration of 5 mM. A second volume of llama seminal plasma (20 ml) was incubated without the presence of pronase E (control) in a shaker water bath at 38°C for 12 hours. Samples were centrifuged at 1500 × g for 5 minutes, and the supernatant was aspirated and stored at -70°C.

Mature non-lactating female llamas (n = 25), ≥ 4 years of age and weighing 100-150 kg, were given 5 mg Armour Standard LH (Lutropin-V) to synchronize follicular wave emergence among animals, as described in Experiment 2. Twelve days after LH administration, llamas with a follicle ≥ 8 mm in diameter were assigned randomly to 2 groups (n = 10 per group) in which 2 ml of pronase-treated seminal plasma or 2 ml of control seminal plasma was given by intramuscular injection. Llamas were examined daily by transrectal ultrasonography until Day 2 (Day 0 = treatment) to detect ovulation, and again on Day 8 to detect the presence of a CL.

### Statistical analyses

Ovulation rate was compared among groups by Fisher's exact test in all experiments. The diameter of the dominant follicle before treatment was compared among groups by analysis of variance in Experiments 1 and 2, and by student's t-test in Experiment 3. CL diameter was compared among groups by student's t-test in the Experiment 1 and by analysis of variance in Experiment 2.

## Results

### Experiment 1 - molecular mass cut-off

The diameter of the largest follicle at the time of treatment did not differ (P = 0.9) among treatment groups and ovulation was observed in only those treated with the ≥ 30 kDa fraction or whole seminal plasma (Table 1). No ovulations were detected in llamas given lower molecular mass fractions of seminal plasma or in those given PBS (negative control). The diameter of the CL on Day 8 (Day 0 = treatment) did not differ between groups treated with the ≥ 30 kDa fraction or whole seminal plasma (Table 1).

**Table 1:** Bioactivity of different molecular mass fractions of llama seminal plasma in female llamas (Experiment 1).

	≥ 30 kDa (n = 9)	10-30 kDa (n = 9)	5-10 kDa (n = 9)	< 5 kDa (n = 9)	WSP (n = 9)	PBS (n = 9)
Follicle diameter* (mm)	9.5 ± 0.7	9.3 ± 0.6	9.8 ± 0.7	10.0 ± 0.7	9.3 ± 0.5	11.3 ± 0.8
Ovulation (%)	9/9^a^ (100%)	0/9^b^ (0%)	0/9^b^ (0%)	0/9^b^ (0%)	9/9^a^ (100%)	0/9^b^ (0%)
CL diameter on Day 8* (mm)	10.1 ± 0.5	----	----	----	10.8 ± 0.7	----

Whole seminal plasma (WSP, positive control), phosphate buffered saline (PBS, negative control). Values expressed as mean ± SEM; Day 0 = treatment.

* No difference between groups (P = 0.9).

^a, b^Proportions with different superscripts are different (P < 0.001).

### Experiment 2 - treatment with proteinase K, charcoal, or heat

**Figure 1:**
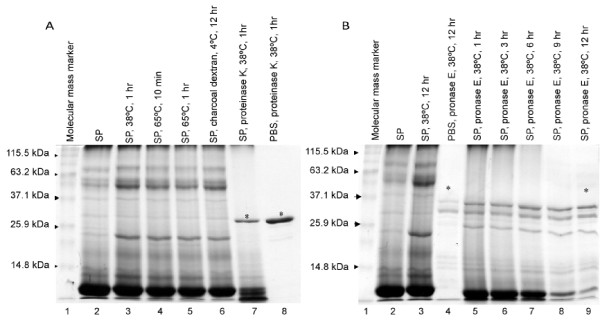
**Llama seminal plasma protein separation on SDS-PAGE after exposure to different treatments in Experiment 2 (A) and Experiment 3 (B)**. Seminal plasma samples were reduced, denatured, and separated on 12% polyacrylamide gel and stained with Coomassie Blue R-250. **A) **Lane 1 was loaded with a molecular mass standard. The remaining lanes were loaded with 20 μg of seminal plasma (SP): Lane 2 - non-treated SP; Lane 3 - SP kept at 38°C for 1 hour; Lane 4 - SP heated to 65°C for 10 min.; Lane 5 - SP heated to 65°C for 1 hour; Lane 6 -- SP treated with charcoal dextran at 4°C for 12 hours; Lane 7 -- SP treated with proteinase K (500 μg/ml) at 38°C for 1 hour; Lane 8 -- phosphate buffered saline (PBS) plus proteinase K (500 μg/ml) at 38°C for 1 hour; * Protein band represents proteinase K. **B) **Lane 1 was loaded with a molecular mass standard. The remaining lanes were loaded with 30 μg of seminal plasma: Lane 2 - non-treated seminal plasma (SP); Lane 3 - SP kept at 38°C for 12 hours; Lane 4 - phosphate buffered saline (PBS) plus pronase E (500 μg/ml) at 38°C for 1 hour; Lane 5, 6, 7, 8, 9 - Seminal plasma treated with pronase E (500 μg/ml) at 38°C for 1, 3, 6, 9 or 12 hours, respectively. * Protein bands represent pronase E.

Ovulations were detected in all the llamas 7/7 (100%) in each treatment group (Table 2). A CL was detected in all females after treatment, and CL diameters did not differ among groups. Gel electrophoresis of seminal plasma treated with heat or charcoal dextran resulted in a similar protein band pattern to that of untreated seminal plasma (Fig. 1). However, treatment of seminal plasma with proteinase K resulted in a markedly different protein band pattern; proteins were rendered to less than 19.4 kDa. The prominent band with a molecular mass of ~30 kDa was identified as proteinase K itself (Fig. 1-A, lanes 7 and 8).

**Table 2:** Bioactivity of llama seminal plasma after treatment with charcoal, proteinase K, or heat in female llamas (Experiment 2).

	Seminal plasma treatment			
	Untreated (n = 7)	Charcoal (n = 7)	Proteinase K (n = 7)	Heat 65°C (n = 7)
Follicle diameter at treatment (mm)*	10.4 ± 0.9	10.8 ± 0.9	9.3 ± 0.2	9.0 ± 0.7
Ovulations*	7/7	7/7	7/7	7/7
CL diameter on Day 8 (mm)*	10.5 ± 0.5	11.3 ± 0.7	11.7 ± 0.6	11.5 ± 0.8

Values expressed as mean ± SEM; Day 0 = treatment.

* No significant differences among groups

### Experiment 3 - treatment with pronase E

Ovulations were detected only in llamas given untreated (control) seminal plasma (Table 3). A CL was detected in all llamas that ovulated in the control group and in none of the llamas given pronase E-treated seminal plasma. Gel electrophoresis of seminal plasma treated with pronase E resulted in a protein band pattern that was markedly different from that of untreated seminal plasma (Fig. 1-B). The band pattern was similar among incubation times (1, 3, 6, 9 and 12 hours), but the degree of enzymatic digestion appeared more complete at the 12-hour incubation period and was, therefore, selected for the purposes of the llama bioassay. The consistent band pattern at the ≤ 37.1 kDa marker in lanes containing pronase E (Fig 1-B, lanes 4 to 9) identified the mixture of proteolytic enzymes constituting pronase E.

**Table 3:** Bioactivity of llama seminal plasma after treatment with pronase E in female llamas (Experiment 3).

	Seminal plasma treatment	
	Untreated (n = 10)	Pronase E (n = 10)
Follicle diameter at treatment (mm)	9.7 ± 0.4^a^	9.0 ± 0.6^a^
Ovulations	9/10^a^	0/10^b^
Maximum CL diameter (mm)	10.8 ± 0.3	----

Values expressed as mean ± SEM.

^a, b^Within rows, values with different superscripts are different (P < 0.01).

## Discussion

The presence of a factor in seminal plasma responsible for eliciting ovulation has been well documented in camelids [2,6,18]. Camelids are species considered to be induced-ovulators, but the ovulation-inducing factor has also been detected in the seminal plasma of species considered to be spontaneous-ovulators [i.e., bull, stallion, boar; [10,11,19]]. To date, the influence of specific components of seminal plasma on ovarian function has not been the subject of systematic examination. Several fractions of Bactrian camel seminal plasma, eluted by ion-exchange chromatography, have been proposed as bioactive components [20,21], but interpretation of the results is limited because of the lack of a validated bioassay to quantitatively test the effects of various fractions. Furthermore, the studies were not designed to examine non-protein constituents of seminal plasma.

The experimental approach used in the present study was aimed at identification through systematic attempts to ablate the biological activity of OIF in seminal plasma. To this end, OIF in llama seminal plasma was subjected to molecular mass filtration to determine approximate size, heat to determine if it is temperature labile, charcoal dextran to determine if it is lipid soluble, and proteases to determine if it is a protein. The bioactivity of fractions of seminal plasma obtained after respective treatments was tested using a validated llama ovulation bioassay [2].

Based on the results of Experiment 1, the molecular mass of OIF present in llama seminal plasma is roughly ≥ 30 kDa, which is consistent with a report on Bactrian camels in which the molecule was suggested to be a folded complex of ≥ 50 kDa [20]. Initial attempts to inactivate OIF in the present study (Experiment 2), however, were unsuccessful and results document the robust nature of OIF, in contrast to the labile nature of the factor reported in Bactrian camels [20]. That is, the bioactivity of OIF in seminal plasma was not altered by heat treatment, charcoal dextran extraction, or enzymatic digestion with proteinase K.

Given that the bioactive fraction was larger than a rough cut-off of 30 kDa (Experiment 1), the inability to inactivate OIF with proteinase K (Experiment 2) was unexpected. Despite that seminal plasma proteins were rendered to less than approximately 19 kDa with proteinase K, the bioactivity of seminal plasma remained intact. Because of this inconsistency and that the results of Experiment 2 were inconclusive about the type of molecule involved, a more aggressive protease (pronase E) was chosen in Experiment 3 to determine if the molecule was indeed a protein. Proteinase K is a serine endoprotease [22] but pronase E is a mixture of endo- and exo-proteinases that hydrolyses practically all peptide linkages in proteins and peptides [23]. Treatment of seminal plasma with pronase resulted in more complete rendering of proteins to < 15 kDa compared with proteinase K, and the bioactivity of OIF was ablated. These results provide rationale for the hypothesis that OIF is a protein with more than one bioactive form based on molecular size.

Although not measured in this study, estrogen and prostaglandin were considered unlikely candidates for OIF because 1) their molecular mass (273 Da and 354 Da, respectively) would have placed them in the ≤ 5 kDa fraction of seminal plasma, yet no ovulation or CL development was detected in females given this fraction (Experiment 1), and 2) the bioactivity was not abolished by steroid extraction with charcoal dextran (Experiment 2). The latter is consistent with the conclusion that estradiol does not play a role in eliciting the preovulatory LH surge in induced ovulators [24].

Prostaglandin, present in boar seminal plasma or secreted by the endometrium in response to seminal estrogens, has been implicated in the mechanism of ovulation in gilts [25,26]; however, seminal plasma was given by intrauterine infusion and it is not clear whether the effect on ovulation was mediated directly at the level of the ovary or if it was triggered by prostaglandin-induced luteolysis followed by a preovulatory LH surge. In this regard, seminal plasma was given intramuscularly in the present study to minimize the potential confounding effects of physical stimulation of the genital tract and endogenous production of prostaglandin. Local prostaglandin, as an ovulation-inducing factor, is also inconsistent with the collective results of three previous studies in which 0/42 llamas and alpacas ovulated after intrauterine deposition of phosphate buffered saline with or without concomitant curettage of the endometrium [2,5]. Furthermore, unlike prostaglandin, treatment with OIF was associated with an immediate surge in circulating LH concentrations; i.e., plasma LH concentrations in female llamas increased within 15 minutes and peaked within 2 hours of intramuscular administration of llama seminal plasma [2].

The hypothesis that OIF is a GnRH-like molecule is inconsistent with the findings of a study in which the LH-releasing effect of seminal plasma on rat pituitary cell culture was not suppressed when anti-GnRH antibodies were added to the culture media [9]. Furthermore, by direct comparison in vivo, OIF elicited a more sustained LH surge than GnRH [2]. Lastly, GnRH is a decapeptide with a molecular mass of 1.18 kDa - far below the bioactive fraction identified in Experiment 1 - and unlike OIF, GnRH is sensitive to proteolytic degradation (endopeptidases) within minutes [27].

We conclude that OIF in llama seminal plasma is a protein molecule with a molecular mass of roughly ≥ 30 kDa. The factor is robust in that the bioactive component was resistant to enzymatic digestion with proteinase K, and incubation at 38°C for 12 h or at 65°C for 1 hour. Only enzymatic digestion with pronase E abolished the ovulation-inducing activity of llama seminal plasma.

## Competing interests

The authors declare that they have no competing interests.

## Authors' contributions

MR participated in designing the study, acquisition, analysis and interpretation of data, and in writing and revising the manuscript. WH participated in acquisition and interpretation of the data. As senior author, GA provided the intellectual impetus for the study, and contributed to the experimental design, data acquisition, analysis and interpretation, as well as writing and revising the manuscript. All authors read and approved the final manuscript.

## References

[B1] Mann T Biochemistry of semen and of the Male Reproductive Tract 1964 Butler & Tanner Ltd, Frome UK 493

[B2] Adams GP Ratto MH Huanca W Singh J Ovulation-inducing factor in the seminal plasma of alpacas and llamas Biol Reprod 2005 73 452 457 10.1095/biolreprod.105.040097 15888733

[B3] England BG Foot WC Matthews DH Cardozo AG Riera S Ovulation and corpus luteum function in the llama (lama glama) J Endocrinology 1969 45 505 513 10.1677/joe.0.0450505 5366111

[B4] Fernandez-Baca S Madden DHL Novoa C Effect of different mating stimuli on induction of ovulation in the alpaca J Reprod Fertil 1970 22 261 267 5464117 10.1530/jrf.0.0220261

[B5] Ratto MH Huanca W Singh J Adams GP Local versus systemic effect of ovulation-inducing factor in seminal plasma of alpacas Reprod Biol Endocrinology 2005 3 29 10.1186/1477-7827-3-29 PMC1190216 16018817

[B6] Chen BX Yuen ZX Pan GW Semen induced ovulation in the Bactrian camel (Camelus bactrianus) J Reprod Fertil 1985 73 335 339 10.1530/jrf.0.0740335 3900379

[B7] Sokol RZ Peterson M Heber D Swerdlof RS Identification and partial characterization of gonadotropin-releasing hormone-like factors in human seminal plasma Biol Reprod 1985 33 370 374 10.1095/biolreprod33.2.370 3899202

[B8] Izumi I Makino T Iizuka M Immunoreactive luteinizing hormone-releasing hormone in the seminal plasma and human semen parameters Fertil Steril 1985 43 617 620 3886437 10.1016/s0015-0282(16)48506-7

[B9] Paolicchi F Urquieta B Del Valle L Bustos-Obregon E Biological activity of the seminal plasma of alpacas: stimulus for the production of LH by pituitary cells Anim Reprod Sci 1999 54 203 210 10.1016/S0378-4320(98)00150-X 10066107

[B10] Ratto MH Huanca W Singh J Adams GP Comparison of the effect of ovulation-inducing factor (OIF) in the seminal plasma of llamas, alpacas, and bulls Theriogenology 2006 66 1102 1106 10.1016/j.theriogenology.2006.02.050 16630652

[B11] Bogle OA Ambati D Davis RP Adams GP Evidence for the presence of ovulation inducing factor in porcine and equine seminal plasma Reprod Fertil Dev 2009 21 101 10.1071/RDv21n1Ab2

[B12] Jochle W Current research in coitus-induced ovulation: A review J Reprod Fertil Supplement 1975 22 165 207 810583

[B13] Wabersky D Südhoff H Hahn T Jungblut PW Kallweit E Calvete JJ Ensslin M Hoppen HO Wintergalen N Weitze KF Topfer-Petersen E Advanced ovulation in gilts by the intrauterine application of a low molecular mass pronase-sensitive fraction of boar seminal plasma J Reprod Fertil 1995 105 247 52 8568767 10.1530/jrf.0.1050247

[B14] Adams GP Griffin PG Ginther OJ *In situ *morphologic dynamics of ovaries, uterus and cervix in llamas Biol Reprod 1989 41 551 558 10.1095/biolreprod41.3.551 2686763

[B15] Ratto MH Huanca W Singh J Adams GP Comparison of the effect of natural mating, LH, and GnRH on interval to ovulation and luteal function in llamas Anim Reprod Sci 2005 91 299 306 10.1016/j.anireprosci.2005.03.015 15896931

[B16] Ratto MH Singh J Adams GP Ovarian follicular wave synchronization and pregnancy rate after fixed-time natural mating in llamas Theriogenology 2003 60 1645 1656 10.1016/S0093-691X(03)00176-6 14580647

[B17] Laemmli UK Cleavage of structural proteins during the assembly of the head of bacteriophage T4 Nature 1970 227 680 685 10.1038/227680a0 5432063

[B18] Xu YS Wang HY Zeng GQ Jiang GT Gao HY Hormone concentrations before and after semen-induced ovulation in the Bactrian camel (Camelus bactrianus) J Reprod Fertil 1985 74 341 346 4045809 10.1530/jrf.0.0740341

[B19] Bogle OA Ratto MH Adams GP Prepubertal mouse bioassay for ovulation-inducing factor (OIF) in seminal plasma Reprod Fertil Dev 2008 20 190 10.1071/RDv20n1Ab221

[B20] Pan G Chen X Liu D Li D Xie Q Ling F Fang L Isolation and purification of the ovulation-inducing factor from seminal plasma of the Bactrian camel (Camelus bactrianus) Theriogenology 2001 55 1863 1879 10.1016/S0093-691X(01)00528-3 11414491

[B21] Xilong L Zhao X Separation and purification of ovulation-inducing factors in the seminal plasma of the Bactrian camel (Camelus bactrianus) Vet Res Communication 2004 28 235 245 10.1023/B:VERC.0000017370.74401.be 15074769

[B22] Betzel C Pal GP Struck M Jany DD Saenger W Active-site geometry of proteinase K. Crystallographic study of its complex with a dipeptide chloromethyl ketone inhibitor FEBS LETTERS 1986 197 105 110 10.1016/0014-5793(86)80307-6 3512298

[B23] Shyh-Haur Y Chien-Hou WU Wann-Yin L Chemical modification of aminopeptidase isolated from pronase Biochem J 1994 302 595 600 8093013 10.1042/bj3020595 PMC1137269

[B24] Bakker J Baum MJ Neuroendocrine regulation of GnRH release in induced ovulators Frontiers in Neuroendocrinology 2000 21 220 262 10.1006/frne.2000.0198 10882541

[B25] Kotwica J Mechanism of prostaglandin F-2 alpha penetration from the horn of the uterus to the ovaries in pigs J Reprod Fertil 1980 59 237 241 7190608 10.1530/jrf.0.0590237

[B26] Claus R Physiological role of seminal components in the reproductive tract of the female pig J Reprod Fertil Supplement 1990 40 117 131 2192032

[B27] Handelsman DJ Swerdloff RD Pharmacokinetics of gonadotropin-releasing hormone and its analogs Endocrine Reviews 1986 7 95 105 10.1210/edrv-7-1-95 3007081

